# Super-Clausius–Clapeyron scaling of extreme precipitation explained by shift from stratiform to convective rain type

**DOI:** 10.1038/s41561-025-01686-4

**Published:** 2025-04-28

**Authors:** Nicolas A. Da Silva, Jan O. Haerter

**Affiliations:** 1 Complexity and Climate, Leibniz Centre for Tropical Marine Research, Bremen, Germany; 2 Department of Physics and Astronomy, University of Potsdam, Potsdam, Germany; 3 Niels Bohr Institute, University of Copenhagen, Copenhagen, Denmark; 4 School of Science, Constructor University Bremen, Bremen, Germany

**Keywords:** Projection and prediction, Atmospheric dynamics, Climate change

## Abstract

Short-duration precipitation extremes pose a risk to human lives and infrastructure and may be strongly affected by climate change. In the past two decades, several studies reported that extreme rainfall intensity can increase with temperature at rates exceeding the thermodynamic Clausius–Clapeyron rate. Two explanations have been proposed for this: (1) convective precipitation—arising from thunderstorms—might be strongly invigorated with temperature; (2) a statistical shift from low-intensity stratiform rainfall to higher-intensity convective rainfall might amplify the scaling rate with temperature. Here we use high spatio-temporal-resolution lightning records in Europe to test these two hypotheses at the storm scale, that is, within 5 km spatially and 10 min temporally. We show that the statistical shift in rain type alone accounts for the observed super-Clausius–Clapeyron scaling rate, and when considered in isolation, both stratiform and convective precipitation extremes increase at the Clausius–Clapeyron rate—thus refuting hypothesis (1). Mesoscale convective systems, which play a dominant role in generating precipitation extremes, do feature a super-Clausius–Clapeyron scaling rate because of a substantial increase in their convective fraction with dew point temperature above 14 °C. Analyses of intensity–duration–frequency curves show that extreme sub-hourly storms are the most strongly intensified with higher dew point temperatures.

## Abstract (web summary)

Warming-induced changes in extreme precipitation intensity that exceed the Clausius–Clapeyron scaling rate can be explained by a statistical shift from stratiform to convective precipitation, according to an analysis of lightning records across Europe.

## Main

Understanding the mechanisms leading to short-duration precipitation extremes is critical in assessing societal impacts, such as those resulting in flash floods^1–3^, and the potential frequency increase of such events in a warmer climate^4–7^. A robust finding in climate models is that relative humidity is nearly constant in future climate projections^8–10^. If so, specific humidity will vary with saturation vapour pressure as given by the Clausius–Clapeyron (CC) relation. A long-standing hypothesis is that precipitation extremes might increase at a rate close to the approximate 7% K^−1^ predicted by the CC relation for typical near-surface temperatures^8,9^.

However, short-duration precipitation extreme scaling rates exceeding the CC rate were reported for several mid-latitude^11–14^ and tropical regions^15,16^ and even replicated in certain simulations^17,18^. Other work^19–21^ cautioned that an apparent super-CC scaling could be the result of a statistical shift in precipitation type from weak stratiform precipitation—resulting from large frontal precipitation bands at low temperatures—to intrinsically more intense thunderstorm precipitation at higher temperatures.

Yet the inherent mechanisms underlying convection were suggested to be capable of bringing about such disproportionate scaling^22^. Indeed, conditioning on times and regions with predominantly convective activity, super-CC scaling of precipitation extremes was described in several areas^12–14,23^. These findings were interpreted in a way that, beyond possible statistical effects, the super-CC scaling of extreme precipitation might indeed have a physical, mechanistic origin^7^.

All observational studies reporting super-CC scaling of extreme convective precipitation discriminated convective from stratiform precipitation at a relatively low spatio-temporal resolution on the order of a few hours and hundreds of kilometres (refs. ^12–14,23^). More specifically, Berg et al. (2013)^23^ used 3-hourly surface synoptic observations of cloud types at approximately 50-km spatial resolution to classify convective and stratiform precipitation over Germany. Related work defined convective precipitation on a precipitation event basis^12^, selecting precipitation events for which at least one lightning strike was detected during the event and within 30 km of the station location. Ivancic and Shaw (2016)^13^ defined convective precipitation similarly, namely as precipitation occurring during a day with lightning activity reported at a maximal distance of 32 km from their station locations. Park and Min (2017)^14^ adopted a method similar to that of Berg et al. (2013)^23^, using the most dominant observed cloud type during a 3-h time window to determine the precipitation type. However, convective and stratiform precipitation types often occur at much higher spatio-temporal variability and the two types generally coexist within the same precipitation system^24^. In particular, long-lived mesoscale convective systems (MCSs) typically span several hundred kilometres in diameter^25^, dominate extreme rainfall yield over Europe^26^, contain both stratiform and convective subregions^27^ and are projected to increase in intensity and frequency over Europe^28^.

Here we call into question that a physical mechanism causing the super-CC scaling exists. From our lightning-based classification of convection, which we pair with high-resolution dew point temperature and precipitation observations over Germany, we conclude that super-CC increases have a purely statistical origin. We, however, describe situations where such statistical superposition may nonetheless have practical relevance, such as during MCS passages or in intensity–duration–frequency (IDF) curves.

## Detecting convective precipitation at high resolution

**Fig. 1:**
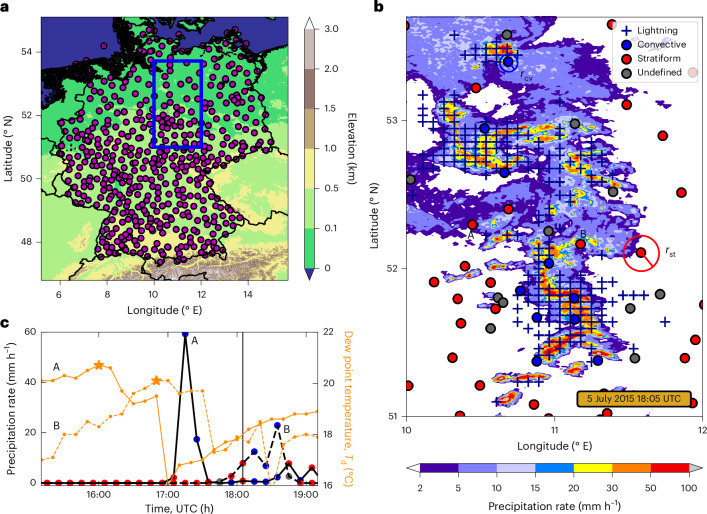
Separating convective and stratiform precipitation. **a**, In situ weather stations used in the analysis (filled magenta circles), recording 10-min precipitation and dew point temperature, super-imposed on an elevation map, which was created from the General Bathymetric Chart of the Oceans 2021 topography dataset (note the colour bar). The blue box highlights the subregion enlarged in **b**. **b**, Snapshot of radar-detected rainfall intensities (colour bar) for the subregion highlighted in **a** on 5 July 2015 at 18:05 UTC. Crosses indicate lightning occurrence between 18:00 and 18:10 UTC. Blue- and red-filled circles represent station locations with lightning occurrence within a distance *r*_cv_ = 5 km, classified as ‘convective’, and without lightning occurrence within a distance *r*_st_ = 30 km, classified as ‘stratiform’, respectively. The data from the remaining weather stations (grey-filled circles) are not defined as belonging to either of these categories. Labels A and B refer to example time series in **c**. **c**, Example precipitation time series (black) for stations indicated in **b** by corresponding symbols (solid for A, dashed for B), with colour markers representing classifications into convective, stratiform and undefined records. Orange curves show corresponding dew point temperature time series (right vertical axis). Orange stars highlight maximum dew point temperatures relevant for the precipitation records at 18:10 UTC (grey vertical line) (Methods).

**Extended Data Fig. 1:**
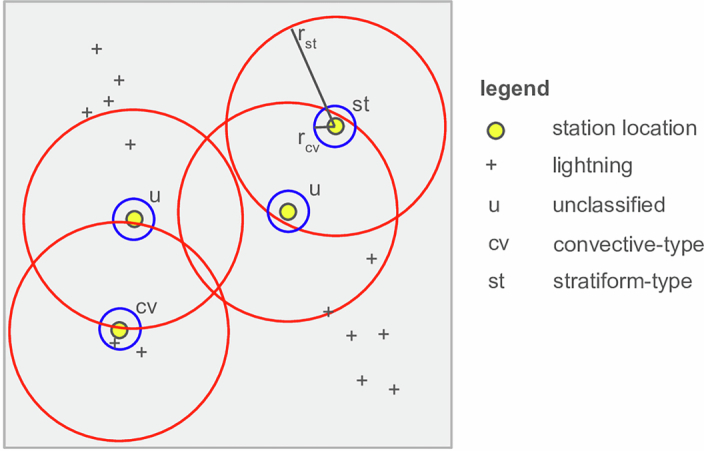
Schematic describing the classification procedure. Station locations (yellow), lightning occurrences (plus-symbols) and rainfall types, as described in legend. The radii of *r*_*st*_ (large red circles) and *r*_*cv*_ (small blue circles) are indicated in the schematic for one case. Note that one st-type station exists, where no lightning was detected within a circle of radius *r*_*st*_, one cv-type station exists, where lightning occurred within a circle of radius *r*_*cv*_, and two u-type stations exist, where lightning existed within *r*_*st*_ but not within *r*_*cv*_ from the stations (*Details*: Methods).

**Extended Data Fig. 2:**
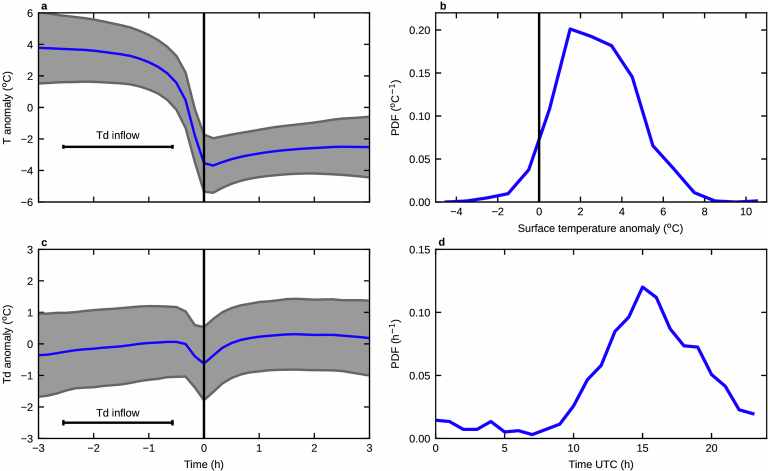
Statistics for 10-minute extreme convective precipitation events. Averaged time series of (**a**), surface temperature (T) anomaly, and (**c**), surface dew point temperature (*T*_*d*_) anomaly around 10-minute extreme convective precipitation periods (black vertical line, at t=0). The anomalies were computed from a 6-hour mean centred on the 10-minute extreme precipitation time. The grey shading areas indicate the spread of the anomalies (plus and minus one standard deviation). The horizontal segment is centred on the mean *T*_*d*_ retained for the analysis and has a width of two standard deviations. Normalised histograms (PDF) of, (**b**), T anomalies at the point in time of the retained *T*_*d*_, and (**d**), of the hour (UTC) when the 10-minute extreme convective precipitation occurs. The black vertical line in (**b**) indicates the 0^∘^C anomaly.

We base our analysis on a large station network (514 stations) covering Germany (Table 1 and Fig. 1a). Both the accumulated precipitation and the dew point temperature (*T*_d_) are recorded at 10-min intervals, in total representing 7,405 years of aggregated data between 2005 and 2020. We combine this large amount of data with the similarly high-resolution (0.071° × 0.045° on a longitude–latitude grid at 10-min temporal intervals) EUropean Cooperation for LIghtning Detection (EUCLID) lightning dataset to discriminate convective precipitation at an unprecedented degree of precision (Table 1). We define convective-type precipitation as accumulated precipitation during a 10-min window (*τ*_cv_ = 10 min) during which at least one cloud-to-ground (CG) lightning flash was detected within a radius *r*_cv_ = 5 km of the station location. Conversely, we define stratiform precipitation as 10-min accumulated precipitation for which no CG lightning flash was detected within a (variable) radius *r*_st_ of the station location and within a *τ*_st _= 3-h window centred on the starting time of the precipitation record. We take the type of the remaining precipitation data as uncertain and assume that it may contain both convective and stratiform precipitation (Methods and Extended Data Figs. 1 and 2).

**Table 1:** Summary of datasets used

Dataset	Temporal resolution	Time range	Spatial coverage
Stations (DWD)^44^	10 min	2005–2020	514 stations
Lightning (EUCLID)^45,46^	10 min	2005–2020	0.071° × 0.045°
Radar (RADOLAN, DWD)^47^	5 min	2007–2019	1 km × 1 km
Topography (GEBCO)		2021	15”

Columns indicate the name of each dataset, its temporal resolution, the time range within which the data were analysed and the spatial coverage or resolution, respectively (Methods).

The EUCLID lightning flashes generally coincide with the highest precipitation intensities (Fig. 1b), confirming their utility in identifying convective cells. Exploiting this method, we are able to cleanly separate convective from stratiform precipitation in convective environments such as during the occurrence of MCSs (Fig. 1b,c). During these events, convective precipitation may be of short duration, surrounded by stratiform precipitation and preceded by peaks in *T*_d_ fuelling the precipitation system (Fig. 1c). We match each 10-min precipitation accumulation with the maximum *T*_d_ in the 3-h time window preceding the 10-min precipitation record. This procedure allows us to derive the scaling of 10-min precipitation extremes with *T*_d_, denoted *P*_99_(*T*_d_). *P*_99_(*T*_d_) for a given *T*_d_ bin is thereby defined as the 99th percentile of all 10-min intervals with accumulated precipitation exceeding a threshold of 0.1 mm (Methods).

## Scalings of precipitation extremes

**Fig. 2:**
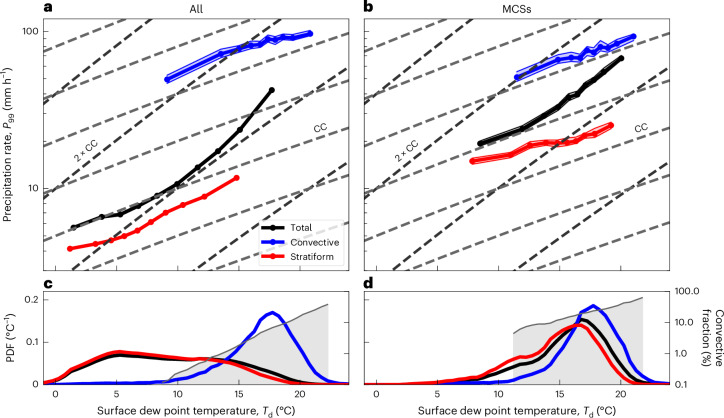
Scaling of precipitation extremes with surface dew point temperature. **a**, *P*_99_ (*T*_d_) for rainfall classified as convective (blue), using *r*_cv_ = 5 km, stratiform (red), *r*_st_ = 300 km and *τ*_st_ = 3 h, and all precipitation (black). Data are presented as the 99th percentile with 95% confidence intervals (shadings) estimated using a non-parametric method based on the binomial distribution and its normal approximation for large sample sizes (Methods). Dashed light and dark grey curves indicate 1 × CC and 2 × CC rates (Methods). **b**, Analogous to **a** but conditional on rainfall within MCSs using *r*_cv_ = 10 km. For the stratiform type, *r*_st_ = 30 km and *τ*_st_ = 10 min were used. The black curve represents all precipitation within MCSs, irrespective of lightning flashes. **c**, Normalized histograms (PDF, probability density functions) for each of the three types shown in **a**. Shaded grey area indicates convective fraction (right vertical axis; note the logarithmic scale). **d**, Analogous to **c** but conditioned on precipitation within MCSs. The convective fraction curves shown in **c** and **d** increase at rate of ≈ 41% °C^−1^ and ≈ 25% °C^−1^ (respectively) with *T*_d_. Note the logarithmic vertical axis scaling in **a** and **b** and **c** and **d**, right axis. Source data

When enforcing a strict separation between convective and stratiform precipitation, that is, *r*_cv_ = 5 km, *τ*_cv_ = 10 min, *r*_st_ = 300 km and *τ*_st_ = 3 h, we find that extreme convective precipitation in fact increases with dew point temperature according to the CC relation. Similarly, *P*_99_(*T*_d_) for stratiform precipitation is close to the CC relation but with a reduction of precipitation intensities by a factor of eight compared to *P*_99_(*T*_d_) for convective precipitation (Fig. 2a). This factor between convective and stratiform precipitation intensity is consistent with the observed order of magnitude difference in updraught vertical velocities between convective and stratiform clouds^29^.

Figure 2c shows the occurrence frequencies of convective, stratiform and total precipitation as a function of *T*_d_. Consistent with previous work^19^, stratiform precipitation dominates at low *T*_d_, peaking near 5 °C, whereas convective precipitation peaks at higher *T*_d_ (≈ 18 °C). In fact, the contribution of convective precipitation increases approximately exponentially (at a rate *β* ≈ 41% °C^−1^) from 7 °C to 22 °C. Thus, the gradual shift from weak stratiform to heavy convective precipitation with increasing *T*_d_ generates a purely statistical super-CC (nearly 2-CC) scaling of extreme total precipitation.

Although much less dramatic, the slight super-CC behaviour of extreme stratiform precipitation may be related to the same statistical effect as our definition of stratiform precipitation may still include a small portion of convective precipitation resulting from convective systems that do not produce any CG lightning^30^. Whereas the slope for the stratiform type is consistent with previous studies, the non-super-CC behaviour of extreme convective precipitation reveals that the much more discriminate spatio-temporal definition of convective precipitation used here may be required to obtain accurate scaling of convective precipitation extremes with *T*_d_.

**Extended Data Fig. 3:**
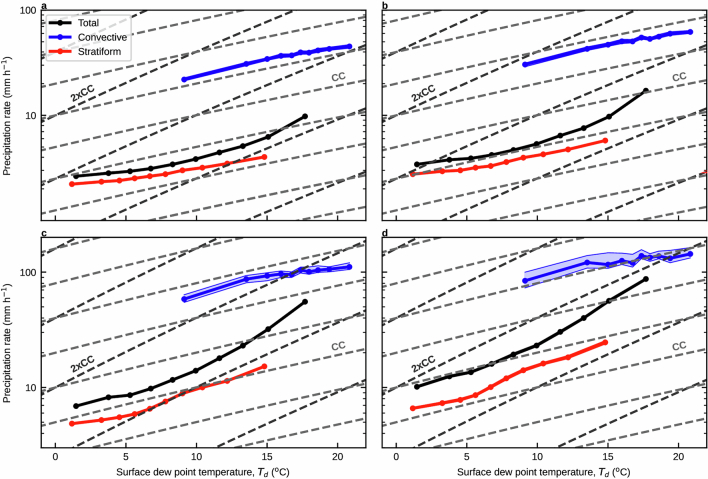
Sensitivity to percentile in the definition of extremes. Analogous to Fig. 2a but using the 90th (**a**), 95th (**b**), 99.5th (**c**), and 99.9th percentiles (**d**), thus modifying the percentile used to define extreme precipitation. **Data are presented as the 90th (respectively 95th, 99.5th, 99.9th) percentile with 95% confidence intervals (shadings) estimated using a non-parametric method based on the binomial distribution and its normal approximation for large sample sizes** (***Details***: **Methods**). Note the change in y-axis range between panels **a**, **b** and panels **c**, **d**.

**Extended Data Fig. 4:**
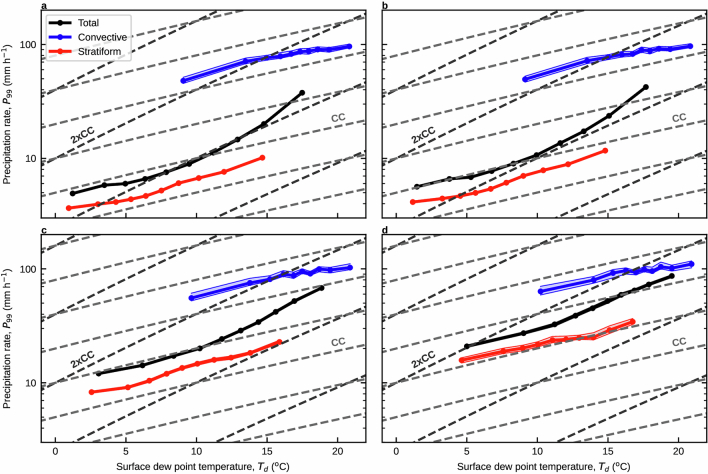
Sensitivity to the precipitation threshold. Analogous to Fig. 2a but selecting 10-minute precipitation records exceeding 0.05 mm (**a**), 0.1 mm (**b**), 0.5 mm (**c**), and 1 mm (**d**), thus modifying the precipitation threshold. **Data are presented as the 99th percentile with 95% confidence intervals (shadings) estimated using a non-parametric method based on the binomial distribution and its normal approximation for large sample sizes** (***Details***: **Methods**).

**Extended Data Fig. 5:**
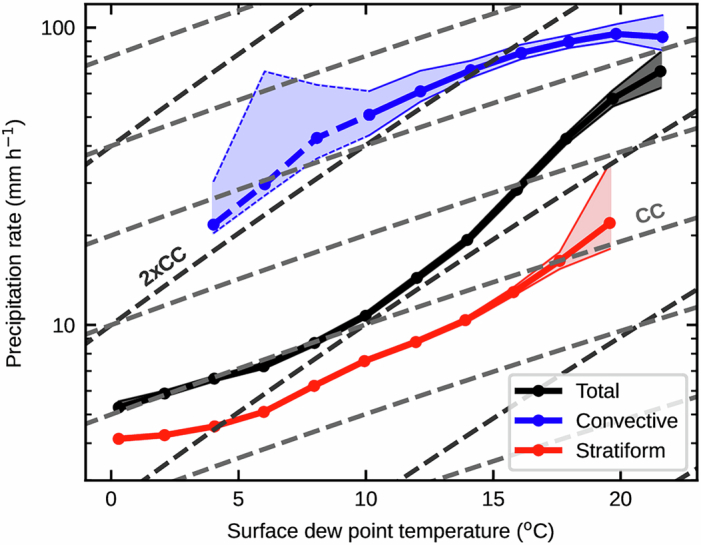
Scaling for fixed bin widths. Analogous to Fig. 2a but using fixed *T*_*d*_ bin widths. The curve for convective precipitation extremes below *T*_*d*_ < 10^∘^C is unreliable (see the error bars) and is therefore represented in a dashed blue line on an indicative basis. **Data are presented as the 99th percentile with 95% confidence intervals (shadings) estimated using a non-parametric method based on the binomial distribution and its normal approximation for large sample sizes** (***Details***: **Methods**).

To ensure that these findings on a simple-CC scaling of convective extremes with *T*_d_ do not depend on our particular choice of parameters, we systematically modify the percentile used for the definition of extremes (Extended Data Fig. 3), the threshold used for defining non-zero precipitation records (Extended Data Fig. 4) and compute the scalings for fixed *T*_d_ bin widths (Extended Data Fig. 5). In either case, the scaling of convective-type precipitation intensity remains within simple-CC scaling.

**Extended Data Fig. 6:**
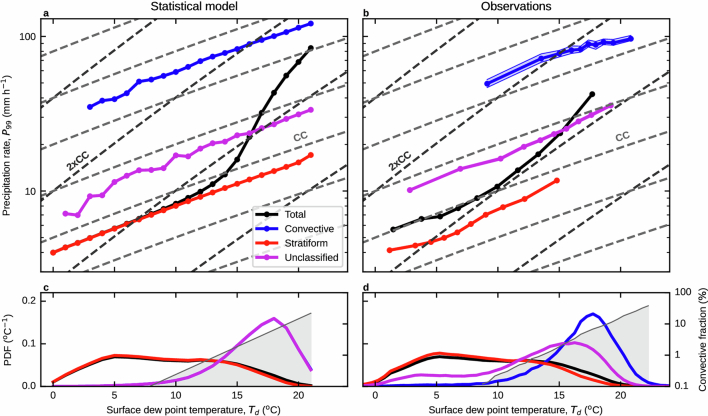
Scaling for the statistical model. Analogous to Fig. 2 but for the statistical model (**a, c**) and observations (**b, d**; corresponding to Fig. 2a,c). We additionally show the scalings and normalised histograms of the unclassified events (magenta). The magenta and blue curves coincide in panel **c**. Allowing for stronger clustering at high dew point temperatures in our simple model would separate the magenta and blue curves in panel **c**, but would only produce little changes for the slope of the unclassified precipitation intensity with dew point temperature (magenta curve in **a**). **Data are presented as the 99th percentile with 95% confidence intervals (shadings) estimated using a non-parametric method based on the binomial distribution and its normal approximation for large sample sizes** (***Details***: **Methods**).

**Extended Data Fig. 7:**
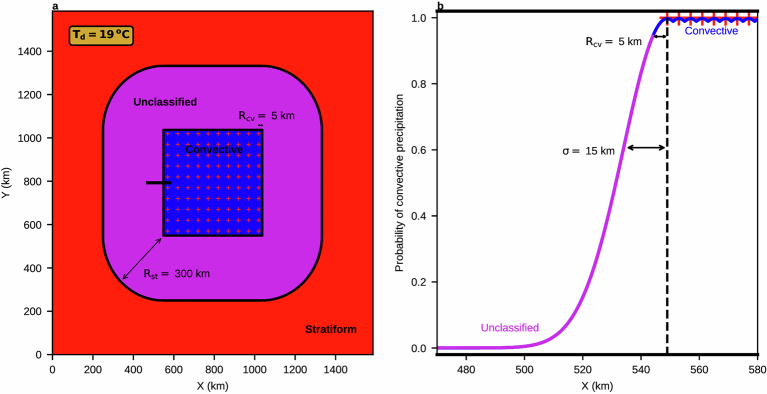
Spatial distributions in the statistical model. Stratiform (red), unclassified (magenta), and convective (blue) areas as defined from a densely filled spatial pattern (here chosen square for simplicity) of lightning strikes (symbolically represented by red crosses) defined in the statistical model, here for the case of *T*_*d*_ = 19^∘^C (**a**). Slice, corresponding to the thick black segment at Y = 800 km in a, of the spatial probability distribution of convective precipitation assumed in the statistical model (**b**). The probability decreases with the distance from the closest lightning strike (red crosses) as a Gaussian with a standard deviation set as *σ* = 15 km. The blue and magenta segments of this probability distribution are consistent with the location of the convective and unclassified categories (respectively). The dashed black vertical line is indicative of the location of the closest lightning strike for all X < 549 km (*Details*: Methods).

Due to the above strict criteria for separating convective from stratiform precipitation, about half of the precipitation events remain unclassified. We find that extreme precipitation from these unclassified events also scale as nearly CC at intermediate values between convective and stratiform extreme precipitation rates (Extended Data Fig. 6b,d). Devising a conceptual model where both convective and stratiform precipitation scale as CC (Methods and Extended Data Fig. 7), and applying our detection method to the generated statistical data, we show that the CC scaling of unclassified events (Extended Data Fig. 6a,c) is indeed possible under the underlying CC assumptions for both convective and stratiform precipitation.

## Sensitivity to the definition of convective precipitation

**Fig. 3:**
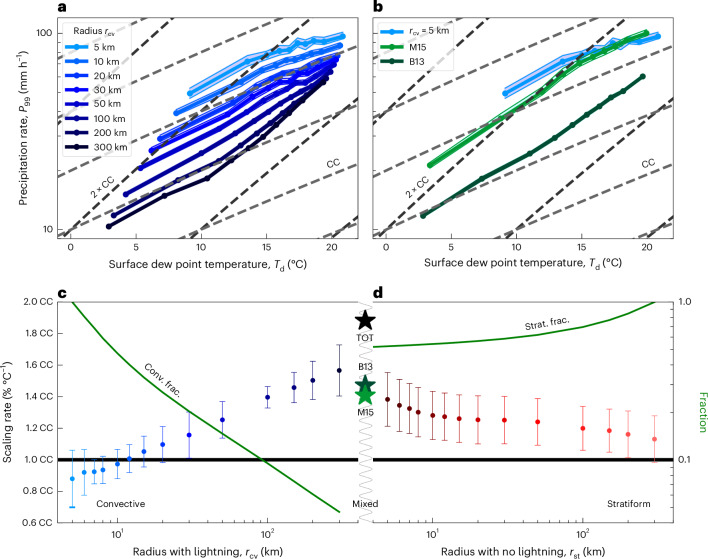
Sensitivity of extreme scaling to detection radius. **a**, Analogous to Fig. 2a but for varying detection radius *r*_cv_ (legend shows colour coding). **b**, Statistical consistency with previously published super-CC results: M15^12^, using a maximum intensity criterion over a precipitation event; mimicking B13^23^, using *r*_cv_ = 50 km and *τ*_cv_ = 3 h; the light-blue curve is a replication of the *r*_cv_ = 5 km curve shown in **a** (Methods). **c**, Mean slopes of the curves in **a** (calculated over 10 data points), which correspond to decreasing values of *r*_cv_, in shades of dark to light blue. Purely convective cases lie to the left of the plot, that is, small *r*_cv_. The green curve indicates the convective fraction (Conv. frac.) corresponding to each *r*_cv_. **d**, Analogous to **c** but for *r*_st_, that is, purely stratiform cases lie to the right of the plot. The green curve indicates the stratiform fraction (Strat. frac.) corresponding to each *r*_st_. Star symbols between panels **c** and **d** indicate the mean slopes for all precipitation (TOT; Fig. 2a, black curve), and M15 and B13 (shown in **b**). Note the logarithmic vertical axis scaling in **a**, **b** and **c** (right axis) and the logarithmic horizontal axis scaling in **c** and **d**. Data in **c** and **d** are represented as mean values ± an error estimate based on the mean of the squared residuals from a linear fit to 10 data points (Methods). Source data

One may ask which resolution is high enough to quantify the scaling of convective-type precipitation extremes. To explore this, we now systematically vary the resolution by adjusting the radius *r*_cv_ within which CG lightning is detected. Successful detection of CG lightning within an area of radius *r*_cv_ during a time interval *τ*_cv_ = 10 min qualifies this area as convective type. Allowing *r*_cv_ to range from 5 km to 300 km while keeping *τ*_cv_ = 10 min, we plot the curve *P*_99_(*T*_d_) for various values of *r*_cv_ (Fig. 3a). Visually, the curves, displayed using a logarithmic vertical scale, show a systematic increase in slope as the radius is increased, that is, when the condition on convection is made more lenient. For the smallest available *r*_cv_ = 5 km, the dependence on *T*_d_ is very close to a CC increase. For larger *r*_cv_, the curves become steeper, especially at the higher values of *T*_d_, where systematic exceedence of the CC rate is visually apparent.

To summarize this systematic steepening, we derive the mean rate of increase for each of the curves. We observe a gradual increase of the mean slopes from approximately 0.9 CC for *r*_cv_ = 5 km to approximately 1.5 CC for *r*_cv_ = 300 km (Fig. 3b). The increase is such that the mean slope can no more be qualified as CC for radii exceeding 20 km (at the 95% confidence level).

To further strengthen our point, we also recomputed and plotted the scaling of extreme convective precipitation as defined by Molnar et al. (2015)^12^ and Berg et al. (2013)^23^ (Fig. 3c), showing that the coarse spatio-temporal definition of convective precipitation indeed results in the statistical super-CC rate found in these previous studies. We thereby mimic the scaling of Berg et al. (2013)^23^ by selecting 10-min precipitation accumulations for which at least one CG lightning flash was detected within less than 50 km—representing a rough mean of the surface synoptic observation spatial resolution in Germany—over a 3-h time window centred on the respective 10-min precipitation time point. A comparison of the scaling found in Berg et al. (2013)^23^ with the corresponding scaling at 10-min temporal resolution shows that coarser temporal definition of convective precipitation may further enhance the super-CC scaling.

Analogously, we also evaluate the effect of increasing the radius *r*_st_ used in the definition of stratiform precipitation and observe a gradual decrease of the mean slopes from approximately 1.5 × CC at 5 km to approximately CC at 300 km (Fig. 2b). These findings underscore that unambiguous separation of convective-type from stratiform-type precipitation requires very strict conditioning on lightning, where lightning must occur within the immediate vicinity, that is, few km and less than 10 min, from a precipitation measurement for the convective type. For unambiguous separation of the stratiform type, lightning must not occur in the mesoscale vicinity. Otherwise, the statistical mix of types will appear as a super-CC increase.

**Extended Data Fig. 8:**
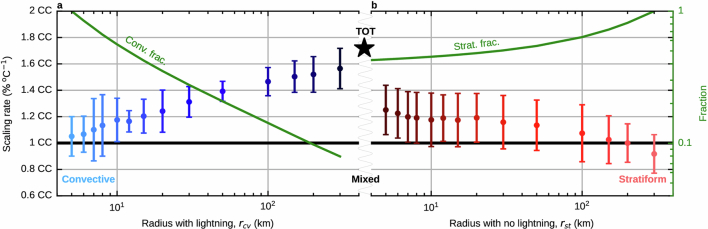
Sensitivity of extreme scaling to the detection radius for hourly accumulations. Analogous to Fig. 3c,d but for hourly precipitation. **Data in a and b are represented as mean values** +/ − **an error estimate based on the mean of the squared residuals from a linear fit to 10 data points** (***Details***: **Methods**).

**Extended Data Fig. 9:**
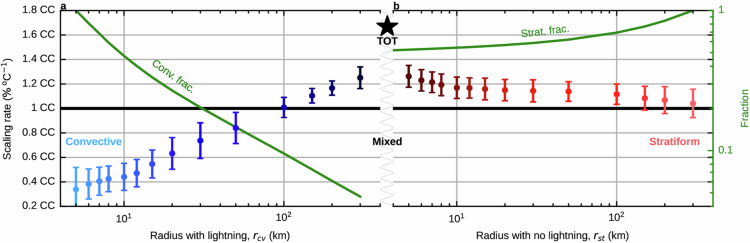
Sensitivity of extreme scaling with temperature to detection radius. Analogous to Fig. 3c,d but replacing dew point temperatures by temperatures. **Data in a and b are represented as mean values** +/ − **an error estimate based on the mean of the squared residuals from a linear fit to 10 data points** (***Details***: **Methods**).

Repeating this analysis using hourly accumulated precipitation—a common accumulation interval in climate models—shows similar behaviour but a higher (<10 km) spatial resolution is required to properly separate convective from stratiform precipitation and obtain a CC scaling (Extended Data Fig. 8). Consistent with earlier studies^7,12,31^, the scaling rates derived from air temperatures are significantly lower than when using dew point temperatures, although comparable resolution dependence exists (Extended Data Fig. 9).

## Scaling for individual storms

The above analysis makes the point that neither convective- nor stratiform-type precipitation extremes exceed the CC rate when taken by themselves. Thus, there is no detectable mechanistic effect by which either of the two types intensifies more rapidly than expected from thermodynamics. Yet precipitation impacts society also as a statistical blend of these two precipitation types, in particular during times when MCSs pass over metropolitan areas. For MCSs, changes in the statistical contribution from convective versus stratiform sub-areas may well affect their precipitation footprint generated over human settlements—making the difference between flooding or not.

Using a recent algorithm for detecting MCSs over Europe^26^, we therefore revisit the question of extreme rainfall scaling, now restricting to areas within MCS. The overall finding is that MCSs contain a blend of convective- and stratiform-type precipitation, but the individual CC-like scaling still holds for either of the two types alone (Fig. 2b). Taking both types together, a clear super-CC scaling is found, thus indicating that MCSs, as a whole, deliver disproportionately more intense rainfall as *T*_d_ increases. This increase is explained by examining the proportion of convective versus stratiform-type precipitation within MCSs as *T*_d_ is varied (Fig. 2d): larger *T*_d_ makes the occurrence of convective-type precipitation more likely, even when conditioning on MCSs. Thus, a given MCS, taken as an entity, is expected to yield much more intense rainfall at higher *T*_d_, in accordance with a super-CC increase of MCS mean intensity.

**Fig. 4:**
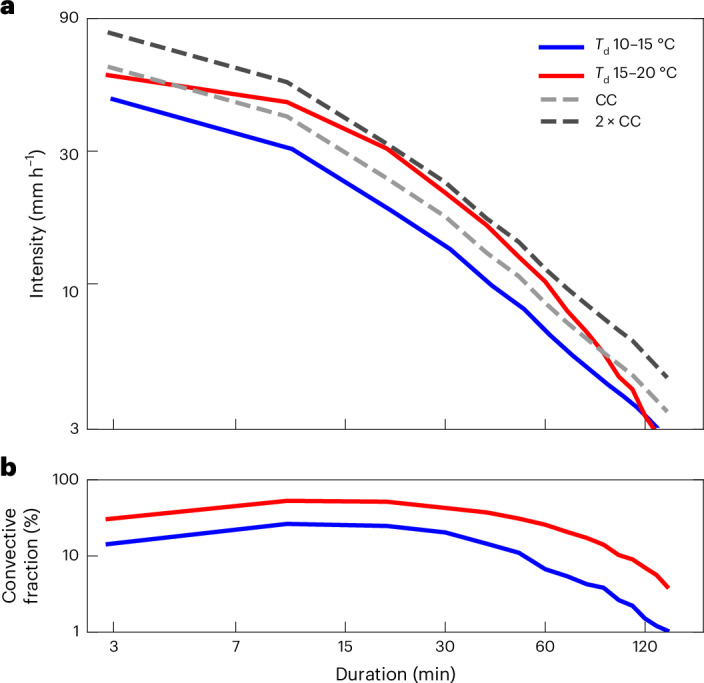
Scalings for storm mean intensities. **a**, IDF curves for the 5-year return period for storms preceded by *T*_d_ between 10 °C and 15 °C (blue) and for storms preceded by *T*_d_ between 15 °C and 20 °C (red). Dashed light and dark grey curves indicate 1 × CC and 2 × CC rates relative to the blue line. **b**, Averaged convective fraction as a function of storm duration for each of the two storm categories shown in **a**. Note the double-logarithmic axis scaling in both panels (Methods). Source data

Analysing all rain events (or storms) perceived by a given fixed observer, we further describe extremes by means of rainfall IDF curves, which are widely used in hydrology to assess flood risk. The obtained IDF curves distinguish storms preceded by high *T*_d_ (15–20 °C) to those preceded by lower *T*_d_ (10–15 °C) (Methods). These curves show an increase in extreme (5-year return period) storm mean precipitation with warming (Fig. 4a). This increase is particularly pronounced and exceeds the CC rate for storm durations of 15–60 min, for which the increase in storm convective fraction (Fig. 4b) has the greatest impact on the storm mean intensity with warming. On the contrary, the storm mean intensity of long-duration (> 60 min) storms only increases at a CC or sub-CC rate. These results were found to be insensitive to the return period when varying it from 1 to 5 years and to the width of the *T*_d_ bins.

## Implications for impact modelling of extreme precipitation

Global warming has been suggested to bring widespread precipitation intensification^6^, in particular regarding convective rainfall extremes^5^. Super-CC increases have served as a possible explanation for such anticipated intensification^4,32^. The current work calls into question if the suggested super-CC increase in convective precipitation extremes actually has a mechanistic base by which thunderstorm cells come with invigorated intensities beyond the thermodynamic rate at higher temperatures^11,22,23^. The present data unambiguously show that there is no exceedence of the CC rate at the scale of individual convective cells. To the contrary, the CC rate is a robust predictor of the change in convective precipitation intensity with temperature. Super-CC changes with temperature are found only as a statistical superposition of distinct rainfall types^19^.

**Extended Data Fig. 10:**
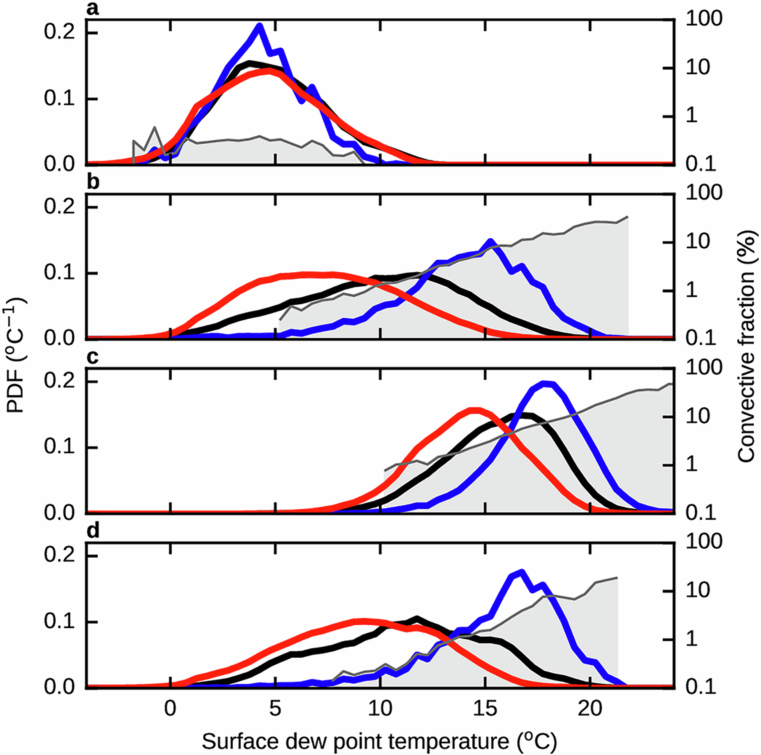
Seasonal dependence of convective fraction. Analogous to Fig. 2c, but for (**a**), winter (DJF), (**b**) spring (MAM), (**c**) summer (JJA), and (**d**) autumn (SON).

Under such statistical superposition, precipitation clusters comprising both convective and stratiform precipitation, such as MCSs^24,26^, could exceed CC scaling as a statistical ensemble. Societal impacts resulting from MCSs, such as flash flooding^2,3^ in increasingly urbanized watersheds^1^, could be amplified under a statistical super-CC scaling. Our analysis based on IDF curves indeed suggests that it is at the flash flood scale, on the order of 15–60 minutes, where storms show strongest super-CC increases. Probing whether convective fraction increases at similar exponential rates in state-of-the-art km-scale climate change simulations would be a logical next step. Such cloud-resolving simulations are also appropriate in detecting thunderstorm events in the spatio-temporal simulation output fields and conditionally analysing the scaling of convective vs stratiform contributions. Precipitation extremes may increase at different rates at different times of the year, as the partitioning into stratiform and convective contributions differs from season to season (Extended Data Fig. 10). Yet, current climate model projections may not accurately reproduce seasonal changes, as showcased by projections that underestimate the inland advection of wintertime maritime systems and the convective fraction of winter storms^33^.

Given the current findings, refocusing the target of extreme precipitation modelling might be useful: with convective and stratiform components individually scaling along the thermodynamic CC rate, the focus could be shifted to unveiling the spatial organization of thunderstorms within mesoscale cloud fields. A strongly clustered thunderstorm population within a given MCS could locally lead to a severe flash flood whereas a scattered population would give rise to moderate, more widespread precipitation at the mesoscale. With the high-resolution simulation data now becoming available^34,35^, detecting changes in clustering with temperature will be feasible. Prominent mechanisms for thunderstorm self-organization, such as cold pool interactions, are now heavily studied, often in idealized settings^36–41^. Conceptual understanding, gained from such works, should find its way into realistic regional-scale studies to help inform future changes in organized convection, for example, convective processes within winter storms and large slow-moving summer systems, which may be more frequent in a warmer climate^33,42,43^.

## Methods

### Observational data

We use the meteorological station dataset from the German Weather Service (Deutscher Wetterdienst, DWD) observational network^44^. Specifically, we extract 10-min accumulated precipitation and 2-m dew point temperature (*T*_d_) measurements from 514 meteorological stations covering Germany (Fig. 1a) for the period between 2005 and 2020. We only retain data that have undergone routine quality control and correction^48,49^.

The radar data are the Radar online adjustment (RADOLAN,^47^) quality-controlled rainfall rate composite version RY from the DWD. This product combines 17 C-band radars covering Germany resulting in a rainfall dataset at 5-min temporal resolution with 1-km horizontal grid spacing. The rainfall rates are derived from radar reflectivity measurements following a refined radar reflectivity to rain rate relationship for liquid hydrometeors, after clutter removal and corrections accounting for topography^47,50^.

We employ the EUCLID lightning dataset to derive convective and stratiform precipitation at high spatio-temporal resolution^45,46^. To do so we only make use of cloud-to-ground lightning flashes (CGs), for their high (>90%) detection efficiency between 2005 and 2020^45^. The number of CGs is originally provided on a 0.045° × 0.071° (latitude–longitude) grid covering all of Germany within 10-min time windows. The location of the detected CGs is considered accurate within 500 m, which is an order of magnitude more accurate compared to the 5-km threshold used for the definition of convective precipitation. Retaining only CGs, our approach discards convective precipitation not accompanied by CG, which may somewhat affect the scaling of our defined ‘stratiform’ precipitation.

The General Bathymetric Chart of the Oceans (GEBCO) 2021 topography dataset, with a 15-arcsecond resolution, is used in Fig. 1a to assess the location of DWD weather stations relative to topography.

### Extreme precipitation curves and confidence bounds

For each 10-min precipitation measurement exceeding a threshold of 0.1 mm, respectively 0.05 mm, 0.5 mm and 1 mm (Extended Data Fig. 4), we associate a corresponding dew point temperature, *T*_d_, as the maximum *T*_d_ occurring in the three hours preceding the precipitation measurement (Figs. 2 and 3). We further condition on ‘dry’ *T*_d_ records, that is, those without measurable non-zero precipitation records, to retain the *T*_d_ corresponding to the inflow of a given precipitation system rather than its outflow which can be strongly influenced by precipitation evaporation. The pronounced diurnal cycle of the occurrence of 10-min extreme convective precipitation (Extended Data Fig. 2d) could indicate that most of this extreme convective precipitation may be driven by diurnal processes, such as those in the boundary layer and lower free troposphere. If no such dry *T*_d_ can be found in the 3 h preceding a precipitation measurement, the 10-min precipitation record is discarded from the analysis. This method successfully retains *T*_d_ records in the warm sector of extreme convective systems (Extended Data Fig. 2a,b), where the temporal variations of *T*_d_ remain of modest amplitude (<2 °C; Extended Data Fig. 2c), which reduces potential attribution errors. We also discard all 10-min precipitation records that are preceded by temperatures not exceeding 5 °C in the 3 h preceding the precipitation measurement, to exclude potential inconsistencies in the derived *P*_99_(*T*_d_) related to snowfall occurrence. The selected values of *T*_d_ are then corrected to account for the different altitudes of the weather stations, assuming a dry adiabatic lapse rate, to the sea level and a constant specific humidity. After this selection, a total of nearly 137 years of precipitation and *T*_d_ pairs are formed. At this step, further conditioning is made to retain convective, stratiform or MCS precipitation (below).

Once the final selection is made, we distribute the resulting pairs into ten bins sorted by *T*_d_, where each bin contains the same number of samples and where we additionally ensure a minimum of 500 samples per bin. For each bin, we then compute the mean *T*_d_ and associate it with the 99th percentile, respectively, the 90th, 95th and 99.5th percentiles in Extended Data Fig. 3, of 10-min precipitation intensities exceeding the chosen threshold. The ten resulting pairs describe the *T*_d_–precipitation extreme relationship used in this study. We explore the effect of defining fixed *T*_d_ bin widths of 2 °C each on the obtained scaling rates in Extended Data Fig. 5.

Following the non-parametric confidence intervals described in the literature^51^ (equations (13) and (14)), we estimate a confidence interval—at the 95% confidence level—on these precipitation percentiles as follows: extracting a number of samples, *N*, from the true distribution of precipitation, the number of elements that exceed the true *q*th precipitation percentile follows a binomial distribution ${{\mathcal{B}}}_{N,1-q}$. If one assumes that the binomial distribution can be approximated by a normal distribution (because *N* is large), a confidence interval on the *q*th percentile can be derived as:

1 $$\mathrm{Pr}\{q-\alpha \} <\mathrm{Pr}\{q\} <\mathrm{Pr}\{q+\alpha \}$$

2 $$\alpha =1.96\times \sqrt{q(1-q)/N}\,.$$

Here Pr denotes the distribution of the *N* precipitation samples, and the numbers enclosed in curly parentheses represent the percentiles of evaluation. For the case of fixed *T*_d_ bin widths (Extended Data Fig. 5), we further calculate an effective number of samples for deriving the confidence intervals. Indeed, the correlation of the 10-min precipitation records within bins having a low number of samples leads to an important underestimation of the confidence intervals when the total number of samples is used to derive confidence intervals. The effective number of samples is calculated assuming that the correlation coefficient between two 10-min precipitation measurements recorded by two different stations at two different time steps is a linear function of the distance separating these stations and of the time interval between these two measurements. Two precipitation measurements are set as uncorrelated if the time interval or the distance between these two measurements exceeds an hour or 50 km, respectively.

The August–Roche–Magnus approximation for saturated vapour pressure (*e*_sat_(*T*_d_)) is used to derive the Clausius–Clapeyron scaling rates as a function of *T*_d_ (in degrees Celsius):

3 $${e}_\mathrm{sat}({T}_\mathrm{d})=610.94\,\exp \left(\frac{17.625{T}_\mathrm{d}}{243.04+{T}_\mathrm{d}}\right)\,.$$

The sensitivity to the precipitation percentile and threshold is explored in Extended Data Figs. 3 and 4.

To derive the mean slopes of the precipitation-extremes–dew-point-temperature relationships in Fig. 3 and Extended Data Figs. 8 and 9, we fit a straight line to the ten data points using a weighted least squares regression. The weights account for the range of temperature values each point represents, with each point weighted by half the distance between its neighbouring temperature points (with edge points adjusted accordingly). The linear fit is performed on the logarithm of the precipitation values. To account for uncertainty, we calculate error bars from the residuals of the fit. Specifically, we estimate the variance by averaging the squared residuals and use its square root to adjust the slope at both ends of the fitted line, providing upper and lower bounds of the scaling rate.

### IDF curves

The IDF curves were computed by defining storms as a time series of 10-min precipitation measurements that each exceed a threshold of 0.1 mm, allowing for gaps with a maximum duration of 10 min. For each storm, the storm mean precipitation intensity is calculated by dividing the total storm precipitation accumulation by its duration. In this, we assumed that the maximum likelihood that a storm spans *n* time steps of *dt* = 10 min each is reached for storm durations *d* = *n* × *dt* − 10 min. We therefore defined storm durations as *d* = *n* × *dt* − 10 min for storms spanning *n* time steps. As an exception, storms spanning only one time step were given a duration *d* = 2.88 min, which corresponds to the best match between the extrapolated IDF curves towards the lowest storm durations and the storm mean intensity values retrieved using this duration. Storms were divided into two categories (10 °C ≤ *T*_d _≤ 15 °C and 15 °C ≤ *T*_d _≤ 20 °C) according to the maximal *T*_d_ values reached within the three hours preceding their onset and conditioned on ‘dry’ (as defined previously) *T*_d_ records. The dashed light and dark grey lines in Fig. 4 were obtained by multiplying the 5-year return intensity in the lower dew point temperature category by a CC or 2 × CC scaling accounting for the mean dew point temperature differences between the upper and lower dew point temperature category in each duration bin and according to equation (3).

### Classification scheme

We define convective, respectively stratiform, 10-min precipitation using the distances *r*_cv_, respectively *r*_st_, from the nearest EUCLID CG detected within a centred time window *τ*_cv_, respectively *τ*_st_. If a CG is detected within less than *r*_cv_ = 5 km from a station within a centred time window *τ*_cv_ = 10 min, the station precipitation is considered as convective (Extended Data Fig. 1), irrespective of whether lightning occurs outside this radius *r*_cv_. If no CG is detected within less than *r*_st_ = 300 km from a station within a centred time window *τ*_cv_ = 180 min, the station precipitation is considered as stratiform. The remainder of the precipitation, which consists of non-CG precipitation events evolving in a convective environment, is flagged as ‘undefined.’ In the main text, convective fraction is defined as the ratio between the number of 10-min precipitation measurements flagged as convective and the total number of 10-min precipitation measurements.

### MCS detection

To define MCS precipitation, we first identify and track RADOLAN precipitation features (defined as contiguous areas of non-zero precipitation in a four-pixel neighbourhood; PFs) using the algorithm presented in Da Silva and Haerter (2023)^26^ with minor modifications accounting for changes in spatio-temporal resolution as described in the following. Whereas we retained the temporal 5-min RADOLAN outputs, we regridded the RADOLAN spatial precipitation field to a 0.1° grid, which improves the likelihood of spatial overlap and thus the tracking of fast moving precipitation systems. The definition of MCS is similar to that in Da Silva and Haerter (2023), substituting the 30-min with the 10-min CG lightning dataset, which improves the detection accuracy. We also increased the PF detection threshold from 2 mm h^−1^ to 4 mm h^−1^ to account for the change from the spatio-temporally smoothed Integrated Multi-satellitE Retrievals for GPM (IMERG) precipitation field to the instantaneous and local measure of precipitation from RADOLAN. For the same reasons, and to ensure sufficient precipitation and *T*_d_ pair records, the condition on the minimum duration for which an MCS has a diameter that exceeds 100 km was reduced from four to one hour. MCS pixels are thus defined at a 5-min interval on a 0.1° grid. We then consider the station location and the 10-min temporal window of measurement. A station 10-min accumulated precipitation measurement is considered as emerging from an MCS when the regridded 0.1° RADOLAN gridbox containing the station, or any of the four nearest neighbours of this RADOLAN gridbox, is part of an identified MCS PF at any time point within the corresponding 10-min time window, that is, either at *t* = 0, *t* = 5 or *t* = 10 min from the beginning of the 10-min window. As selecting MCS precipitation considerably reduces the precipitation and *T*_d_ pair records, we loosen the criteria for defining convective and stratiform precipitation within MCSs to ensure statistical significance of the scaling derived. Indeed, for the MCS convective and stratiform classification, we use *r*_cv_ = *r*_st_ = 20 km and *τ*_cv_ = 10 min and *τ*_st_ = 10 min, respectively.

### Statistical model

We employ a simple statistical model to discuss the realism and potential implications of our results. The underlying main assumptions of this model are that both convective and stratiform precipitation scale at CC and that the convective fraction increases exponentially at a rate of 41% °C^−1^. We then generate synthetic precipitation data for different dew point temperatures as follows: first, we consider a fixed number of lightning strikes and place them in a square area in the middle of a two-dimensional square domain (Extended Data Fig. 7a). Consistent with the EUCLID dataset, the lightning strikes are separated by a distance of 4 km to one another. Second, we vary the domain size to achieve a targeted lightning density that increases exponentially at a rate of 41% °C^−1^, as found in the observations (Fig. 2a,c). This procedure is numerically equivalent to increasing the number of lightning clusters at a fixed domain size. We use our method based on the distance to the closest lightning strike (Extended Data Fig. 1) to classify all grid points into the convective, unclassified and stratiform categories. Each of the defined convective, stratiform and unclassified categories may contain a mix of convective-type and stratiform-type precipitation. We assume that the probability of finding convective-type precipitation around each lightning strike decreases with the distance from this lightning strike (Extended Data Fig. 7b). Specifically, in our model, the probability that rainfall occurring at a given position is of convective type, given that the nearest lightning strike occurs a distance *r* from it, *P*_conv_(*r*), is given by the Gaussian probability distribution

4 $${P}_\mathrm{conv}(r)=\exp \left(-\frac{{r}^{2}}{2{\sigma }^{2}}\right)$$

where we used *σ* = 15 km to achieve consistency with the observed precipitation intensities of the unclassified data points. Using this spatial probability distribution, we are able to calculate the expected number of grid points with convective precipitation for each category. Dividing by the total number of grid points of each category, we obtain an expected convective fraction for each category. We then generate convective and stratiform precipitation data, assuming that they follow an exponentially decaying distribution with a scale parameter that increases according to the CC relationship. This ensures that the 99th percentile, and, more generally, all percentiles, of the generated convective and stratiform precipitation, follows the CC relationship. To reproduce the observed precipitation intensity differences between convective and stratiform precipitation, we assume a ratio of eight between the scale parameter of convective precipitation and that of stratiform precipitation. We distribute the generated convective and stratiform precipitation in the three categories in accordance with the convective fractions derived from the spatial probability distribution (equation (4)). For each temperature bin (here increasing by steps of 1 °C), we constrain the total number of precipitation data so that the final dew point temperature normalized histogram (black curve in Extended Data Fig. 6c) approaches the one of the observations (black curve in Extended Data Fig. 6d). The resulting extreme precipitation curves show great consistency with those of the observations (Extended Data Fig. 6a vs Extended Data Fig. 6b). This simple model could also be used at the scale of MCSs, by scaling down both the central lightning cluster size and the radius *R*_st_, which would yield similar results.

## Online content

Any methods, additional references, Nature Portfolio reporting summaries, source data, extended data, supplementary information, acknowledgements, peer review information; details of author contributions and competing interests; and statements of data and code availability are available at 10.1038/s41561-025-01686-4.

## Source data

Source Data Fig. 2 Statistical source data.

Source Data Fig. 3 Statistical source data.

Source Data Fig. 4 Statistical source data.

## Data Availability

The meteorological station dataset is available online at https://opendata.dwd.de/climate_environment/CDC/observations_germany/climate/10_minutes/ (DWD)^44^. The RADOLAN precipitation dataset is available at https://www.cen.uni-hamburg.de/icdc/data/atmosphere/samd-ltfd-datasets/hdfd-miub-drnet00-l3-rr.html. The BLIDS/EUCLID lightning data are not publicly available due to privacy restrictions but may be provided upon request. Requests should be sent to BLIDS/EUCLID (contact: w.schulz@ove.at), specifying the intended use and complying with a data-use agreement. Response times typically range up to 2 weeks. The GEBCO 2021 topography dataset can be downloaded at https://www.gebco.net/data_and_products/historical_data_sets/. Source data are provided with this paper.

## Code availability

The code used to prepare data, derive statistics and plot figures is available via GitHub at https://github.com/Nicolas-A-Da-Silva/SuperClausiusShiftRainType/ under the Apache 2.0 license.
